# Macintosh laryngoscope and i-view™ and C-MAC® video laryngoscopes for tracheal intubation with an aerosol box: a randomized crossover manikin study

**DOI:** 10.1186/s40981-021-00455-7

**Published:** 2021-06-26

**Authors:** Toshiyuki Nakanishi, Yoshiki Sento, Yuji Kamimura, Kazuya Sobue

**Affiliations:** Department of Anesthesiology and Intensive Care Medicine, Nagoya City University Graduate School of Medical Sciences, Kawasumi 1, Mizuho-cho, Mizuho-ku, Nagoya, Japan

**Keywords:** Aerosol box, Airway management, COVID-19, Laryngoscope, Tracheal intubation, Video laryngoscope

## Abstract

**Background:**

We tested the hypothesis that the C-MAC® video laryngoscope (C-MAC) with an external display is more useful than the disposable i-view™ video laryngoscope (i-view) with an integrated display or a Macintosh direct laryngoscope (Macintosh) for tracheal intubation with an aerosol box.

**Methods:**

In this randomized, crossover manikin study, we recruited 37 medical personnel with > 2 years of dedicated anesthesia experience from five hospitals. After the three successful intubations within 60 s using each laryngoscope without a box, the participants performed tracheal intubation thrice with each laryngoscope with at least 2-h intervals in a determined order. The primary outcome was the intubation time. The secondary outcomes were success rate, Cormack-Lehane grade, and subjective difficulty scale score.

**Results:**

Thirty-seven personnel (11 women and 26 men) with 12 [5–19] (median [interquartile range]) years of anesthesia and intensive care experience were enrolled. There was no significant difference in the intubation time: 30 [26–32] s for Macintosh, 29 [26–32] s for i-view, and 29 [25–31] s for C-MAC (*P* = 0.247). The success rate was 95–100%, without a significant difference (*P* = 0.135). The i-view and C-MAC exhibited superior Cormack-Lehane grades and lower subjective difficulty scale scores than the Macintosh; however, there were no differences between the i-view and C-MAC.

**Conclusions:**

Rapid and highly successful tracheal intubation was possible with both Macintosh, i-view, and C-MAC on a normal airway manikin in an aerosol box. Improved Cormack-Lehane grade and the ease of performing the procedure may support the use of video laryngoscopes.

**Trial registration:**

UMIN Clinical Trials Registry, UMIN000040269. Registered 30 April 2020.

**Supplementary Information:**

The online version contains supplementary material available at 10.1186/s40981-021-00455-7.

## Background

The outbreak of coronavirus disease 2019 (COVID-19) is an important concern for healthcare providers because the causative agent, respiratory syndrome-corona virus-2 (SARS-CoV-2), is highly contagious, primarily via direct contact or droplet transmission. Tracheal intubation is considered one of the highest-risk procedures because of possible aerosol generation and the need to be in close physical proximity with the patient [1–9].

The “aerosol box” was first conceived by a Taiwanese doctor and was reported effective in preventing widespread dispersion of cough droplets during tracheal intubation [10–12]. This box was also expected to be useful in situations where higher-level personal protective equipment (PPE), such as the medical protective head hood and powered air-purifying respirator, is unavailable [13, 14]. However, some previous reports suggested that tracheal intubation in the box can be challenging because it restricts hand movements [11, 14–16].

Video laryngoscopes are recommended for tracheal intubation in patients with COVID-19 to keep distance from patient’s airway [1–7]. Currently, several types of video laryngoscopes are commercially available; some have an integrated display on the body, while others have an external display. There are some recommendations for the use of video laryngoscopes with an external display for patients with COVID-19 because it allows the healthcare practitioner to maintain a reasonable distance from the patient’s airway [3–5]. On the other hand, disposable video laryngoscopes such as i-view™ (i-view; Intersurgical, Wokingham, UK) may be beneficial for considering the risk of virus contamination. Furthermore, given the short supply of disposables for video laryngoscopes and the possible reduced risk of infection, a Macintosh direct laryngoscope (Macintosh) might be considered for use with an aerosol box [5, 6]. Many patients with COVID-19 who are undergoing tracheal intubation are hypoxemic and require rapid, highly successful procedures; therefore, it is essential to identify the optimal device to use with the box [2, 7].

When using an aerosol box, the box’s seam and barriers (eye-protective PPE and box wall) between the operator’s eyes and the patient’s glottis or integrated monitor of the video laryngoscope may impair the operator’s visibility. Thus, a video laryngoscope with an external display may be more useful than other types of laryngoscopes during tracheal intubation with an aerosol box. We, therefore, designed this study to test the hypothesis that the C-MAC® video laryngoscope (C-MAC; KARL STORZ, Tuttlingen, Germany) with an external display is more useful than the i-view with an integrated display when used with an aerosol box.

## Methods

This prospective, randomized, crossover manikin study was conducted at the Nagoya City University Hospital and the Nagoya City East Medical Center from April 30, 2020, to May 11, 2020. The study protocol was reviewed and approved by the Nagoya City University Graduate School of Medical Sciences and Nagoya City University Hospital Institutional Review Board. This study was registered with the UMIN Clinical Trials Registry (identifier UMIN000040269). After verbally explaining the study flow to the participants and showing the video made for instruction, we obtained written consent for study participation. The patients were not involved in the study. All the methods were performed in accordance with the CONSORT 2010 statement: extension to randomized crossover trials.

We recruited medical personnel who were working in the fields of anesthesia and intensive care without previous experience with aerosol boxes from five hospitals (Nagoya City University Hospital, Nagoya City East Medical Center, Kainan Hospital, Kariya Toyota General Hospital, and Aichi Children’s Health and Medical Center) in Japan. According to the standard recommendations, only experienced physicians should perform tracheal intubation for patients with COVID-19; therefore, our study only included personnel with > 2 years of dedicated anesthesia experience after completion of residency training [1–8]. All participants were familiar with Macintosh and other types of video laryngoscopes such as the McGRATH MAC video laryngoscope (Medtronic, Minneapolis, MN, USA) or Airway Scope (Nihon Kohden, Tokyo, Japan) but had little experience with i-view and C-MAC.

**Fig. 1:**
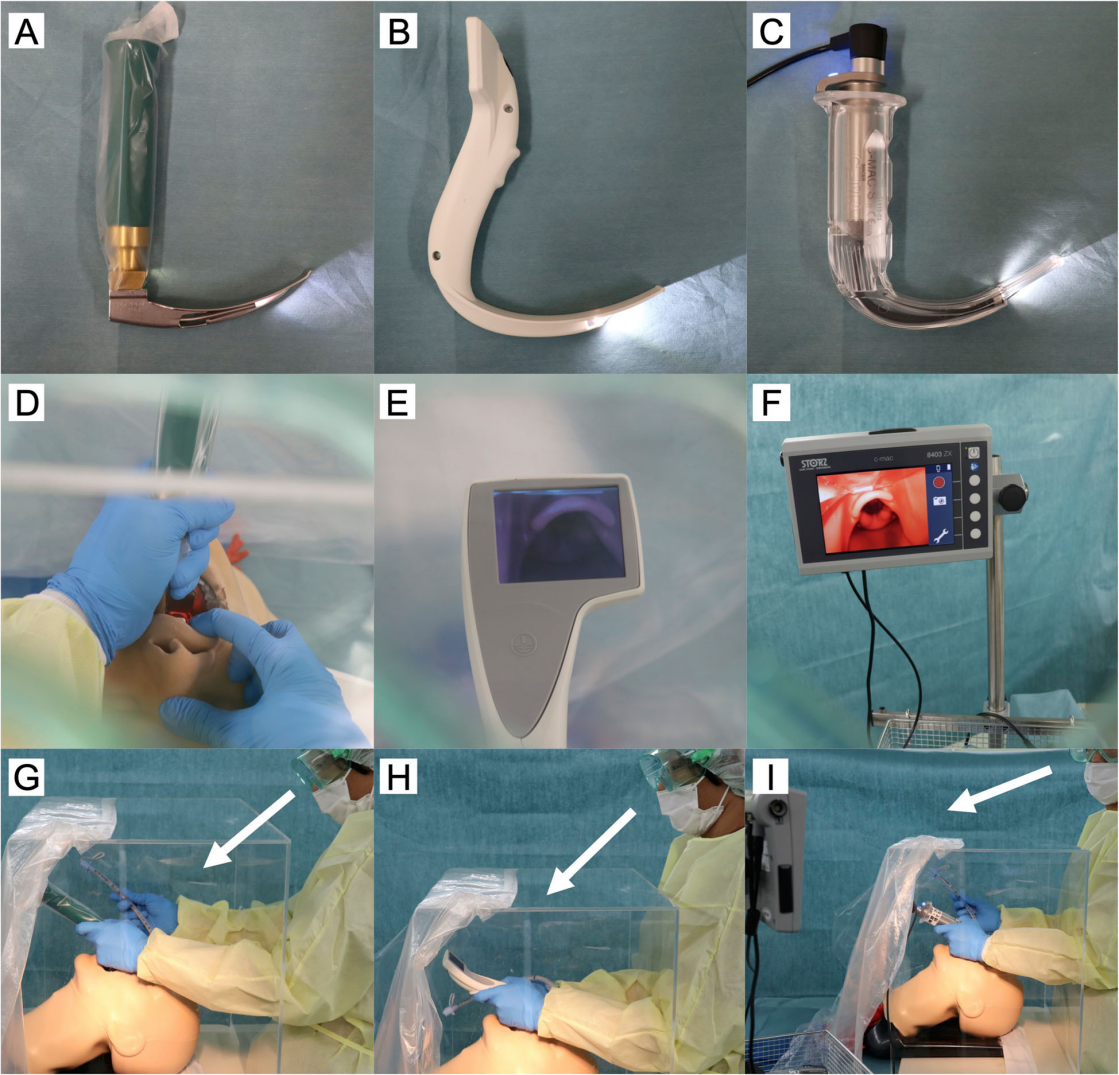
Macintosh, i-view, and C-MAC laryngoscope and visual images of tracheal intubation with an aerosol box. **A** Macintosh direct laryngoscope. **B** i-view video laryngoscope. **C** C-MAC video laryngoscope. **D**–**F** Laryngoscopic view of the glottis in each laryngoscope. **G**–**I** Lateral image during the tracheal intubation in each laryngoscope. The white arrows show the operator’s lines of sight

In this study, we compared the following three types of laryngoscopes: Macintosh, i-view, and C-MAC. The C-MAC is a video laryngoscope with an external display, while the i-view is a display-integrated, one-size-fits-all (equivalent to a Macintosh size 4), single-use video laryngoscope (Fig. 1). A reused size-3 blade was used for Macintosh, and a single-use size-3 Macintosh type blade was used for C-MAC. The AirSim Combo Bronchi X (TruCorp, Lurgan, Ireland) manikin designed for normal airway training was used for all the procedures. A 7.0-mm tracheal tube with a stylet, angled by each participant, was used. Although some arrangements have been reported [16, 17], we created and used an acrylic box based on the original version of the report [10].

Before the main measurement using the box, the participants were trained to familiarize themselves with the three laryngoscopes and the manikin used in the study. The training was conducted in the same manner as the main study, except in the following order (Macintosh, i-view, and C-MAC) without the box until three successful procedures within 60 s with each laryngoscope. All the training and main studies were conducted in an operating room at the two hospitals (Nagoya City University Hospital and Nagoya City East Medical Center). The manikin was placed on the operating table in the supine position under the box. Both the manikin and box were fixed with tape on the table so that the top of the manikin’s head was 10 cm away from the box. During training and the main test, the participating physician wore a long-sleeved gown, double gloves, a surgical mask, face shield or goggles, and a surgical cap. The N95 mask was not used because they are in short supply and have limited influence on intubation procedures. Neither a covering hood nor a powered air-purifying respirator was used. The height of the operating table was adjusted for each participant. In the box, a laryngoscope was placed on the left side of the manikin, and a tray was placed on the right side, where a tracheal tube and a cuff syringe with 8 ml of air were prepared. Six l/min of oxygen was administered to the manikin with a facemask. The participants removed the mask, opened the manikin’s mouth and picked up the laryngoscope. Then the participants picked up a tracheal tube, performed tracheal intubation, and removed the stylet and inflated air into the cuff of the tube by themselves, considering the minimal number of personnel in the operating room. Direct laryngoscopy was used with Macintosh, whereas an indirect (monitor) view was used with i-view and C-MAC for tracheal intubation (Fig. 1). The participants removed the outer glove on their right hand and grasped the reservoir bag for ventilation. The investigators (TN and YS) stood on the right side of the manikin, recording the intubation time with a stopwatch, and helped connect the anesthesia circuit to the tracheal tube. The intubation time was defined as the time between holding the laryngoscope and confirming the first expansion in both lungs. An intubation time > 60 s, esophageal intubation, or single-lung intubation were considered to indicate failure. After each procedure, the participant assessed the Cormack-Lehane grade and the subjective difficulty scale score of tracheal intubation (numeric rating scale 0–10, 0: no difficulty, 10: highest difficulty).

**Fig. 2:**
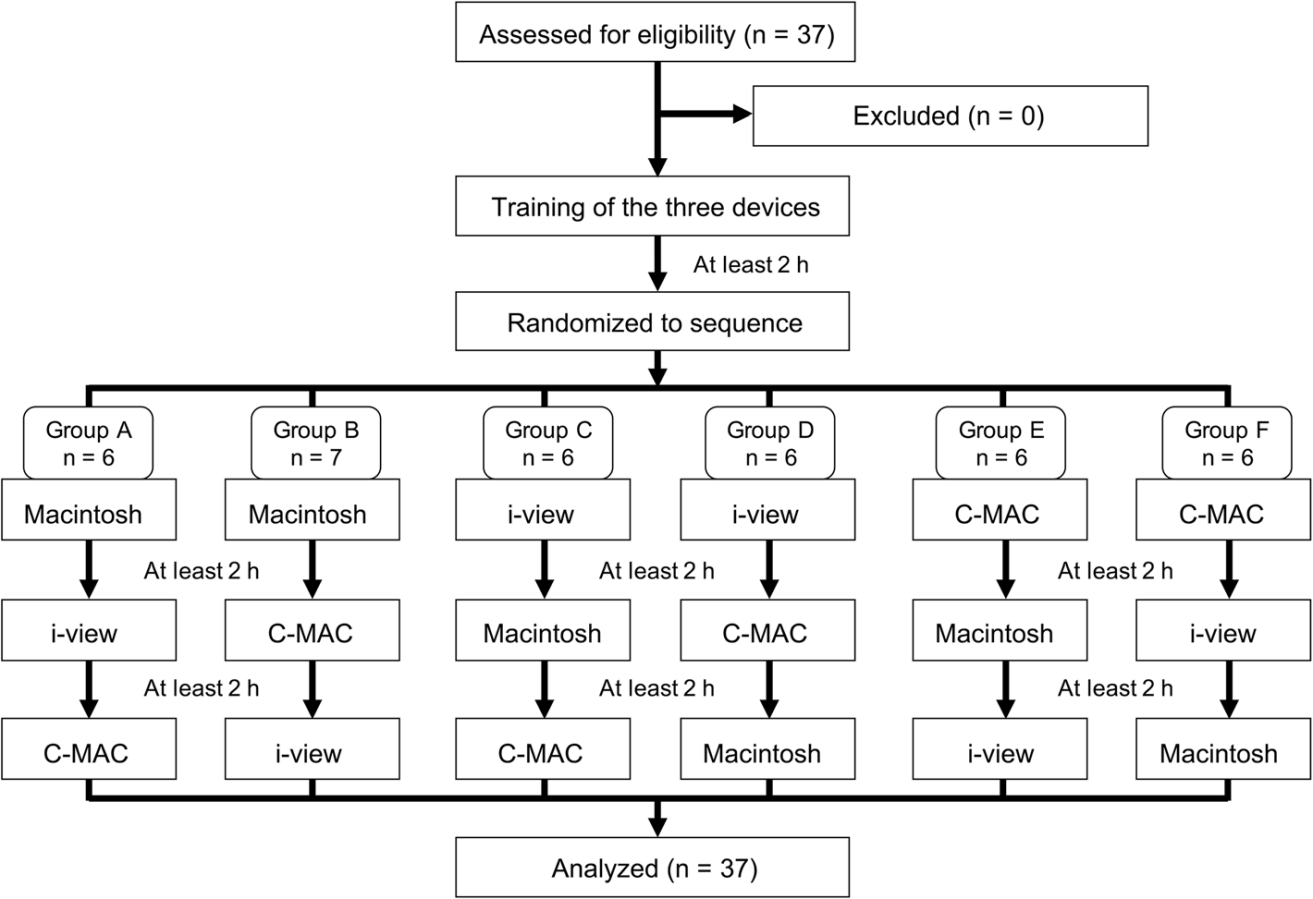
CONSORT diagram of the study participants

At least 2 h after the training, the participants began the main part of the study using an aerosol box. To compare the three laryngoscopes, we used a randomized crossover design by dividing the participants into six groups and testing them in the determined order (Fig. 2). An investigator (TN) who did not participate in the study performed the computer-generated randomization and allocated the participants to the six groups. The participant was blinded to the allocation until immediately before preparing the determined laryngoscope. The participants performed tracheal intubation on the manikin with the box three times by using each assigned laryngoscope. A washout period of at least 2 h was required before the next laryngoscope was used. The primary outcome was the intubation time. The secondary outcome included the success rate, Cormack-Lehane grade, and subjective difficulty scale score.

### Statistical analysis

On the basis of the preliminary analysis performed by investigators who did not participate in the study, we estimated an intubation time of 25 s for C-MAC and 35 s for i-view. With an α error of 1.67% (adjusted for Bonferroni method), power of 90%, SD of 7, and correlation coefficient of 0.5 for 2-tailed statistical analysis, we arrived at a minimum sample size of 10 participants. However, we recruited as many eligible physicians as possible in the study because it also aimed to provide simulation training and increase the secondary endpoint estimate accuracy (success rate).

To compare the intubation time, Cormack-Lehane grade, and subjective difficulty scale score of the three laryngoscopes, we used the median values of the three measurements, considering the learning effect. The intubation time of i-view showed a non-normal distribution (Shapiro-Wilk test *P* < 0.05); the intubation times of the three laryngoscopes are presented as the median [interquartile range (IQR)] values. The Cormack-Lehane grade and subjective difficulty scale score are also presented as median [IQR] values. We used the Friedman test to compare the three devices’ performances. If a significant difference was found, the Wilcoxon signed-rank test with Bonferroni adjustment was used for pairwise comparisons. We compared the proportion of participants who successfully performed all three procedures between the three different laryngoscopes by using the Cochran Q test. All the statistical analyses were performed using the R software (version 3.6.3, R Foundation for Statistical Computing, Vienna, Austria). A *P* value of <0.05 was considered statistically significant.

## Results

We enrolled 37 personnel (11 women and 26 men) with 12 [5–19] years of anesthesia and intensive care experience; all their records were incorporated into the final analysis (Fig. 2).

A summary of the results obtained with the box is shown in Table 1. The intubation time was 30 [26–32] s for Macintosh, 29 [26–32] s for i-view, and 29 [25–31] s for C-MAC, showing no significant difference (*P* = 0.247). The success rate was 95–100% without a significant difference, with two failed attempts with the i-view (one took 66 s, and the other was stopped after 60 s). The Cormack-Lehane grade was lower in i-view and C-MAC than in Macintosh. The subjective difficulty scale score was higher with Macintosh than with i-view or C-MAC. However, there were no differences in the Cormack-Lehane grade and the subjective difficulty scale score between i-view and C-MAC.

**Table 1:** Outcomes using the Macintosh, i-view, and C-MAC laryngoscopes for tracheal intubation in the aerosol box

	A	B	C	A vs. B	A vs. C	B vs. C	*P*
	Macintosh	i-view	C-MAC	*P*	*P*	*P*	
	(*n* = 37)	(*n* = 37)	(*n* = 37)				
Intubation time (s)	30 [26–32]	29 [26–32]	29 [25–31]				0.247
Success	37 (100)	35 (95)	37 (100)				0.135
Cormack-Lehane grade	2 [2–2]	1 [1–1]	1 [1–1]	< 0.001	< 0.001	1	< 0.001
Subjective difficulty scale	4 [3–5]	3 [2–3]	2 [1–3]	< 0.001	< 0.001	0.055	< 0.001

Data are shown as median [interquartile range] or number (percentage). The *P* values of multiple comparisons were adjusted using the Bonferroni method

Detailed results obtained with and without the aerosol box are shown in Supplementary Tables 1 and 2, respectively. In training without the box, the intubation time was 29 [25–33] s for Macintosh, 29 [24–32] s for i-view, and 26 [24–30] s for C-MAC.

## Discussion

In this simulation study that compared the performances of three types of laryngoscopes, namely, Macintosh, i-view, and C-MAC, for tracheal intubation in a manikin with an aerosol box, we found no significant differences in intubation time among the three types of laryngoscopes. All three laryngoscopes facilitated quick tracheal intubation with a 95–100% success rate; however, Macintosh had a higher subjective difficulty scale score and worse Cormack-Lehane grade than i-view and C-MAC.

The present results did not support our hypothesis that the use of C-MAC, which has an external display, is more useful than i-view for tracheal intubation with an aerosol box. This result suggests that skilled personnel may be able to overcome our concerns about visual problems such as the box’s seam and barriers between the eyes and the glottis or monitor (Fig. 1). Recently, Madabhushi et al. enrolled 78 patients with normal airways who had no COVID-19 and found that by using Glidescope (Verathon, Bothell, WA, USA) with an external display, the tracheal intubation time with the aerosol box was non-inferior to that without the box [18]. Furthermore, Puthenveettil et al. reported that C-MAC was easier to use than Macintosh for tracheal intubation with an aerosol box in 60 patients with normal airways without COVID-19 [19]. These results may suggest the potential benefit of an external display when performing tracheal intubation with an aerosol box in real patients [18, 19]. Although an external display might be useful in clinical settings where visual conditions are more unfavorable, such as cloudy or well-worn eye-protective PPE or aerosol boxes, we could not confirm the advantage in this simulation study using a transparent acrylic box.

The subjective difficulty scale score for tracheal intubation was higher for Macintosh than for i-view and C-MAC. In situations where both a Macintosh laryngoscope and a video laryngoscope can be used, our results support using a video laryngoscope because it would facilitate tracheal intubation with the aerosol box. However, video laryngoscopes may not be available because of an insufficient supply of disposable healthcare products [8, 9]. The Macintosh facilitated quick tracheal intubation with a high success rate that was unexpectedly comparable with that of video laryngoscopes, despite the greater difficulty and worse Cormack-Lehane grade. Our study is similar to that of Wakabayashi et al. who found that experienced anesthesiologists did not have clinically prolonged intubation times despite the poorer glottic view when using Macintosh with an aerosol box than without the box [20]. For medical personnel skilled in using Macintosh laryngoscopes, the combined use of a Macintosh laryngoscope and an aerosol box might be an option if a difficult airway is not anticipated and the availability of video laryngoscopes is limited.

Tracheal intubations in the aerosol box were performed with a highly successful rate (95–100%) and median intubation time of 29–30 s, which was not clinically different from the median intubation time of 26–29 s without the box. A recently published meta-analysis revealed that intubation time with an aerosol box increased by 4 s (95% confidence interval 2.4–5.6 s) as compared with that without a box, but this may not be clinically relevant [21]. This meta-analysis also revealed no significant prolongation of intubation time of 1.9 s when the video laryngoscope was used by a consultant. The shorter intubation time in our study may be due to experiences of the participants and their familiarity with the device and manikin through training. The high success rate and short intubation time observed in our study suggest that an aerosol box can be used safely after sufficient training by experienced personnel.

Our study has several strengths. First, we enrolled a moderate number of physicians who had > 2 years of dedicated anesthesia experience and were engaged in airway management at multiple centers. Moreover, the participants in our study wore PPE and underwent tracheal intubation with minimal assistance, referring to recommendations for airway management in patients with COVID-19. Second, we measured the outcomes three times for each laryngoscope and used the median values for the comparisons. In unfamiliarity, one-time measurements may result in long intubation times, which is not clinically meaningful.

Certain limitations of the present study need to be noted. First, this simulation study was performed using a manikin. It is necessary to examine the safety and efficacy of aerosol boxes in clinical practice. Evidence for the use of aerosol boxes in real patients is lacking and must be studied in the near future [21, 22]. Second, differences in the participants’ familiarity with the three laryngoscopes might have affected the results. However, we believe that these effects were minimized because we provided training to the participants before initiating the main study. Third, we did not evaluate physician safety in this study in terms of droplet splash, aerosol spread, and PPE breakage, although no breakage of long-sleeved gowns occurred in our study. Fourth, we used the box with the original design; however, various modifications to the box, with regard to shape and draping, have been reported [10, 16, 17]. These might make it difficult to generalize our study results. Finally, we did not simulate a difficult airway setting in the present study. Furthermore, the participants in our study placed airway management equipment in their optimal position. Our results are not applicable to situations such as difficult airways and emergency airway settings. In such situations, the box should be removed and the airway secured in the best possible manner [11, 12, 17].

## Conclusions

In summary, tracheal intubation in an aerosol box can be performed using any of the following three types of laryngoscopes, namely, Macintosh, i-view, and C-MAC, with an intubation time of 29–30 s and a success rate of 95–100%. Improved Cormack-Lehane grade and ease of procedure may support using a video laryngoscope when using the aerosol box.

## Supplementary Information

**Additional file 1: Supplementary Table 1.** Detailed results using the Macintosh, i-view, and C-MAC laryngoscopes for tracheal intubation in the aerosol box. Supplementary Table 2. Detailed training results using the Macintosh, i-view, and C-MAC laryngoscopes for tracheal intubation without the aerosol box.

## Data Availability

Data are available from the corresponding author on reasonable request.

## Abbreviations

COVID-19: Coronavirus disease 2019
IQR: Interquartile range
PPE: Personal protective equipment
