# Supplementary material for: Macintosh laryngoscope and i-view™ and C-MAC® video laryngoscopes for tracheal intubation with an aerosol box: a randomized crossover manikin study
Source: JA Clin Rep. 2021 Jun 26;7:52. doi: 10.1186/s40981-021-00455-7 (PMC8234758; doi:10.1186/s40981-021-00455-7)
Supplement: Supplementary file 1 — Additional file 1: Supplementary Table 1. Detailed results using the Macintosh, i-view, and C-MAC laryngoscopes for tracheal intubation in the aerosol box. Supplementary Table 2. Detailed training results using the Macintosh, i-view, and C-MAC laryngoscopes for tracheal intubation without the aerosol box. [file 40981_2021_455_MOESM1_ESM.docx]

**Supplementary Table 1**

Detailed results using the Macintosh, i-view, and C-MAC laryngoscopes for tracheal intubation in the aerosol box.

Data are shown as median [interquartile range] or number (percentage).

**Supplementary Table 2**

Detailed training results using the Macintosh, i-view, and C-MAC laryngoscopes for tracheal intubation without the aerosol box.

Data are shown as median [interquartile range] or number (percentage).
